# Chronic spinal cord injury attenuates influenza virus-specific antiviral immunity

**DOI:** 10.1186/s12974-016-0574-y

**Published:** 2016-05-31

**Authors:** Valerie Bracchi-Ricard, Ji Zha, Annalise Smith, Darlah M. Lopez-Rodriguez, John R. Bethea, Samita Andreansky

**Affiliations:** The Miami Project to Cure Paralysis, Department of Neurosurgery, Miller School of Medicine, University of Miami, Miami, FL 33136 USA; Department of Biology, Drexel University, Philadelphia, PA 19104 USA; Department of Microbiology and Immunology, University of Miami Miller School of Medicine, Miami, FL 33136 USA; Department of Pediatrics, University of Miami Miller School of Medicine, Miami, FL 33136 USA

**Keywords:** Spinal cord injury, T cell, B cell, Influenza, Virus, Immunity, Vaccine

## Abstract

**Background:**

Individuals suffering from spinal cord injury (SCI) are at higher risk for respiratory-related viral infections such as influenza. In a previous study (Zha et al., J Neuroinflammation 11:65, 2014), we demonstrated that chronic spinal cord injury caused impairment in CD8^+^T cell function with increased expression of the immunosuppressive protein, programmed cell death 1 (PD-1). The present study was undertaken to establish whether chronic SCI-induced immune deficits would affect antiviral immunity directed against primary and secondary infections.

**Methods:**

Six to seven weeks following a SCI contusion at thoracic level T9, mice were infected intranasally with influenza virus. Virus-specific immunity was analyzed at various time points post-infection and compared to uninjured controls.

**Results:**

We report that chronic thoracic SCI impairs the ability of the animals to mount an adequate antiviral immune response. While all uninjured control mice cleared the virus from their lungs by day 10 post-infection, a significant number (approximately 70 %) of chronic SCI mice did not clear the virus and succumbed to infection-induced mortality. This was attributed to severe deficits in both virus-specific antibody production and CD8^+^ T cell response in injured mice after primary infection. We also determined that previously acquired humoral immunity was maintained after spinal cord injury as vaccination against influenza A prior to injury-protected mice from a homologous viral challenge. In contrast, prior immunization did not protect mice from a heterotypic challenge with a different strain of influenza virus.

**Conclusions:**

Taken together, our data demonstrate that chronic SCI attenuates virus-specific humoral and cellular immunity during the establishment of primary response and impairs the development of memory CD8^+^ T cells. In contrast, B cell memory acquired through vaccination prior to SCI is preserved after injury which demonstrates that antigen-specific memory cells are refractory following injury. Our study defines important parameters of the deficits of chronic SCI-induced immune depression during a viral respiratory infection. Our objective is to better understand the mechanisms of spinal cord injury-induced immune depression with the goal of developing more effective therapies and reduce mortality due to complications from influenza and other infections.

**Electronic supplementary material:**

The online version of this article (doi:10.1186/s12974-016-0574-y) contains supplementary material, which is available to authorized users.

## Background

Central nervous system (CNS) injury such as spinal cord injury (SCI) disrupts the crosstalk between the CNS and the immune system resulting in a syndrome called “CNS Injury-Induced Immuno-depression” (CIDS) characterized by increased susceptibility to infections, worse neurological outcome, and oftentimes death [1, 2]. In fact, more than 50 % of deaths following SCI result from infection [1, 3–5]. Investigations on the effects of SCI on peripheral immune function have yielded valuable insights regarding the negative impact of loss of proper regulation by peripheral nerves on primary and secondary lymphoid tissues. Inflammation and stress responses initiated soon after injury can cause an early deficit in leukocyte number and function [6]. Clinical and experimental models of SCI have validated that during the acute phase of injury, post-SCI both innate and adaptive immunity are severely compromised [6–8] and can persist into the chronic phase [4, 9–11]. While most studies investigating the impact of acute or chronic SCI on immune dysfunction have focused on high thoracic (T3) injury, studies by Held and colleagues have concluded that increased sensitivity to viral infection due to SCI-induced immune depression is level independent [4]. Therefore, it is critical to better understand mechanisms through which SCI mediates systemic immune depression so that complications arising from secondary infections (e.g., chronic hospitalization, worse neurological outcome, and death) can be reduced or alleviated altogether. A number of studies examining SCI-induced immune depression have shown an increase in the susceptibility to microbial infections such as mouse hepatitis virus [4] and *Streptococcus pneumoniae* [12]. However, few have examined the impact of SCI on antiviral immunity using a clinically relevant respiratory virus infection model. For example, SCI patients are at high risk of developing complications of influenza infection followed by secondary pneumonia due to their reduced respiratory function and mobility after injury [3, 13–15]. Influenza A virus is a major respiratory pathogen that causes high morbidity and accounts for a significant number of deaths in both the very young and elderly people (www.cdc.gov). Furthermore, the emergence of new pandemic strains in the past decade have heightened the awareness that immune-compromised patients such as those suffering from SCI are most susceptible to new viruses [16]. In immunocompetent individuals, primary infection generates a robust immunity and requires generation of both virus-specific antibodies and an effector T cell response [17]. This establishes an immunological memory and an immune protection over an individual’s lifespan that can protect against re-infection with the same virus. This response can also be mimicked by proper immunization.

Thus, the goal of this study was to characterize how chronic SCI affects immunity acquired after influenza infection. We used a well-characterized mouse model of influenza virus infection in C57Bl/6J mice [18] to investigate the mechanisms of protective immunity in chronic SCI during primary and secondary viral infections. Intranasal inoculation with type A influenza virus results in a lower respiratory tract infection and induction of both innate and adaptive responses necessary to clear the viral infection. Because of the complex nature of SCI and the finding that high-level injury affects immune function through complete deregulation of the sympathetic nervous system, we chose to investigate SCI-induced immune dysfunction using a low thoracic level (T9) contusion injury model that mostly maintains the central sympathetic regulation to the peripheral lymphoid organs [6, 12]. Six to seven weeks following a thoracic (T9) SCI contusion, mice were intranasally infected with H3N2 influenza A virus. A comprehensive analysis of virus-specific immunity was performed at various time points after infection and compared to uninjured controls. We demonstrate that chronic SCI causes severe morbidity and mortality in mice infected with influenza A virus. Analysis of innate immune gene expression and recruitment of inflammatory cells to the lungs during the initial phase did not show significant differences between uninjured and chronic SCI mice. In contrast, both virus-specific antibody production and CD8^+^ T cell responses were severely compromised in chronically injured mice. Vaccination against influenza prior to injury protected mice from a homologous influenza virus challenge but did protect against infection with a different strain of type A influenza, H1N1 (PR8), pointing to a deficit in CD8^+^ T memory cells. These studies will have broad application to our mechanistic understanding to CIDS and may contribute to novel therapeutic strategies to both improve neurological outcome and reduce death related to immune-mediated complications often seen following CNS injury.

## Methods

### Mice and spinal cord injury

Specific pathogen-free C57BL/6J female mice (3–4 months old and 18–20 g in weight) purchased from Jackson Laboratory were randomized and subjected to laminectomy and subsequent spinal cord injury (SCI) at thoracic level T9 using the Infinite Horizon Impactor at a predetermined force of 70 kDynes, resulting in a severe contusion injury in Animal Biosafety level 1 facility. Immediately after injury, mice were sutured and injected subcutaneously with 1-ml Lactated Ringer’s solution to prevent dehydration and gentamicin (40 mg/kg) to prevent urinary tract infection. Sham controls underwent a laminectomy at T9 without SCI injury. The prophylactic antibiotic treatment was continued for 7-day post-injury. Bladder expression was performed twice daily until recovery of function. By 6- to 7-week post-SCI, mice have usually regained their initial body weight. Uninjured age-matched mice were used as controls (CT). About 1 week prior to infection, mice (SCI, CT, and Sham mice) were transferred to animal biosafety level 2 housing for subsequent influenza infection. All animal protocols were approved by the University of Miami Institutional Animal Care and Use Committee (IACUC) and are in accordance with National Research Council guidelines for the care and use of laboratory animals. We also report compliance with the ARRIVE guidelines as requirement for reporting in vivo animal experiments [19].

### Influenza virus infection

Influenza virus subtypes A/HKx31 (HKx31, H3N2) and A/Puerto Rico/8/34 (PR8, H1N1) were used for these studies. PR8 expresses the surface protein hemagglutinin (HA) and neuraminidase (NA) of H1N1 subtype, whereas the reassorted HKx31 virus expresses the H3N2 surface protein of A/Hong Kong/1/1968 but also contains six internal proteins common to the PR8 virus [20]. H3N2 via intranasal route has intermediate virulence in mice compared to PR8 and infection with either viruses results in complete clearance by day 10 post-infection [18]. Mice were weighed prior to viral infection and then anesthetized with a mixture of ketamine (80 mg/kg) and xylazine (10 mg/kg) mixture. 1 × 10^4^ plaque-forming units (PFU) of HKx31 virus in a 30-μl volume were delivered intranasally. The dose and volume ensure a 100 % rate of infection and mice were monitored daily for weight loss and morbidity. PR8 virus was administered (1x10^4^ PFU) in the secondary infection model for memory response studies.

### Virus titer

Mice were sacrificed as per AVMA (American Veterinary Medical Association) guidelines at various times post-infection. Lungs were harvested, snap frozen in liquid nitrogen, and stored in −80 °C until assayed. Viral titers were determined from homogenized lungs using the 50 % tissue culture infectious dose (TCID_50_) method [21]. Briefly, confluent Madin-Darby canine kidney (MDCK) cells cultured in MEM (Invitrogen) containing 2-mM l-glutamine, 10 μg/ml gentamicin, and 5 % FBS were infected with serial dilution of lung homogenate and incubated at 37 °C for 2 h to allow virus particles to adsorb to cells. The inoculum was washed and replaced with media containing 2 μg/ml of Tosyl Phenylalanyl Chloromethyl Ketone (TPCK)-treated trypsin and incubated for 72 h. Viral infection was scored by observing the cytopathic effect in infected wells after staining with crystal violet and was confirmed by hemagglutination assay with chicken red blood cells.

### RNA extraction and real-time PCR

Lungs were homogenized in TRIzol (Life Technologies) and total RNA isolated following the manufacturer’s protocol. An aliquot of 20 μg of total RNA was further purified using the RNeasy mini kit (Qiagen) with on-column DNase digestion to remove any trace of genomic DNA contamination. cDNA was obtained by reverse transcription of 1 μg of purified RNA using the Omniscript RT kit (Qiagen) and analyzed by quantitative real-time PCR using primer pairs as shown in Additional file 1. For each gene, a standard curve was obtained by diluting defined amounts of the target PCR product. All the data obtained were normalized to β-actin gene expression levels.

### Flow cytometric analysis of innate immune cells in BAL

Mice were sacrificed, and bronchoalveolar lavage (BAL) cells were collected by flushing the exposed trachea after cannulation with an 18-gauge, ½-in. cannula connected to a 1-ml syringe. The BAL cells were harvested by flushing the lungs with 10 % 1XRPMI and centrifugation. The cells were resuspended for flow cytometric analysis in appropriate stains. They were first blocked with anti-CD16/32 (FcR block, eBiosciences) to prevent non-specific staining and then stained with APC Cy7-anti-F4/80 (clone BM8, Biolegend), PE Cy7-anti-CD11b (clone M1/70, eBioscience), APC-anti-CD11c (clone N418, Biolegend), and FITC-anti-Gr1 (clone RB6-8C5, BD Pharmingen) and gated according to Hall et al. [22]. Briefly, cells were first gated as F4/80^+^ or F4/80^−^ cells. F4/80^+^ cells that are CD11b^high^ were referred to as macrophages and those that are CD11c^high^/CD11b^low^ as alveolar macrophages. DCs were gated as F4/80^low^ CD11c^high^ and monocytes as F4/80^low^ CD11c^low^CD11b^mid^. Finally, neutrophils were gated as F4/80^low^ CD11c^low^CD11b^high^ and Gr1^high^. Data were collected using the LSRII flow cytometer and analyzed with FACSDiva 6.1 software (BD Biosciences).

### ELISA for virus-specific antibody

Sera were collected by arterial bleed, and BAL supernatants were harvested from the lungs after flushing once with 0.5 ml 10 % 1xRPMI followed by centrifugation at 1000 rpm for 5 min. Samples were frozen at −80 °C for virus-specific antibody assays. Influenza-specific antibody levels in sera and lung washes were determined by ELISA [23] with an inactivated influenza HKx31, A/Aichi/68 (H3N2) virus (Charles River Laboratories). Briefly, the virus was inactivated with 0.5 % Triton X-100 and coated on plates overnight at 4 °C. Next day, the plates were washed with PBS/0.05 % Tween 20 (Sigma) and blocked with 1 % BSA in PBS for 1 h at room temperature. Serial dilutions of BAL and serum samples were added to the wells and incubated for 2 h at room temperature. After extensive washes, influenza-specific antibodies were detected with the corresponding HRP-conjugated goat anti-mouse IgG, IgM, or IgA fraction (1:1000, Southern Biotechnology Associates). Color development was performed with TMB (3,3′,5,5′-tetramethylbenzidine) substrate (Sigma) for 60 min at room temperature and read at 450 nm in an ELISA reader (Molecular Devices).

### B cell activation assay

Lymphocytes were isolated from spleens as previously described [11]. Mature resting B cells were isolated by depletion of CD43^+^ cells using CD43 (Ly-48) MicroBeads according to the manufacturer’s protocol (Miltenyi Biotec). B cells (10^6^/well) were cultured in 1 ml of complete RPMI (RPMI 1640, 5 % FBS, 100 U/ml penicillin, 100 μg/ml streptomycin) in a 24-well plate with 5 ng/ml of recombinant mouse IL-4 (Gibson) and 5 μg/ml of anti-mouse IgM (Jackson ImmunoResearch Laboratories) for 24 h. B cells cultured in 1-ml complete RPMI were used as the unstimulated control. Both stimulated and unstimulated B cells were harvested and washed with FACS buffer and were incubated with 5 μg/ml Fc receptor block (anti-mouse CD16/32, Biolegend) for 5 min on ice. Cells were then stained with antibodies including APC-efluor780-anti-B220 (eBiosciences, clone HIS24, 1:200), PE-anti-CD86 (Biolegend, clone GL-1, 1:200), APC-anti-MHC class II (eBiosciences, clone M5/114.15.2, 1:1000), and PE/Cy7-anti-CD40 (Biolegend, clone 3/23, 1:50). Cells were detected using LSRII and analyzed by FACS-Diva Version 6.1.3 software (BD Biosciences).

### Virus neutralization assay

Serum samples diluted twofold were mixed in equal volumes with 100 TCID_50_ of HKx31 (H3N2) for 2 h at 37 °C in a 5 % CO_2_ atm. The virus/serum mixtures were then incubated on MDCK cell monolayers in 96-well plates for another 2 h. The cells were washed and maintained in TPCK-containing medium, and infected wells were identified by hemagglutination assay after 3 days.

### Tetramer and intracellular cytokine assay

The kinetics and magnitude of the virus-specific CD8^+^ T cell responses were analyzed by flow cytometry. Spleens were harvested and processed according to published methods [24]. Lymphocytes were isolated from spleens after dissociation and subjected to red blood cell lysis with ammonium-chloride-potassium (ACK) buffer (Invitrogen). Virus-specific CD8^+^T cells were quantified by staining cells for 1 h at room temperature with H-2D^b^ MHC Class I (C57Bl/6J mice) tetramers [25] conjugated to either PE or Alexa488 D^b^NP366, ASNENMETM [26] or PE or APC labeled D^b^PA224, SSLENFRAYV [27] obtained from the NIH Tetramer Facility (Emory University, Atlanta). The cells were washed extensively with FACS buffer (PBS, 1 % BSA, 0.05 % azide) and were surface stained with FITC-conjugated anti-CD8α (clone 53-6.7, BD Pharmingen). For intracellular cytokine assays, lymphocytes were cultured in 96-well round-bottom plates for 6 h at 37 °C in the presence of brefeldin A (Enzo LifeScience) with either 1-μM NP366 or 1-μM PA224 peptide. The cells were next stained with FITC-labeled anti-CD8 (clone 53-6.7) for 30 min and fixed. Intracellular cytokines were detected with PE IFNγ (clone XMG1.2, BD Pharmingen), APC TNF (clone MP6-XT22, BD Pharmingen), and PE-Cy7 granzyme B (clone NZGB, eBiosciences) after permeabilization. Cells were resuspended in FACS buffer, and data was acquired with LSR II (BD Biosciences). A total of 10,000 CD8^+^ T cells were analyzed using FlowJo software (Tree Star) for calculating the numbers of virus-specific CD8^+^ T cells.

### Vaccine study

Mice were vaccinated with inactivated HKx31 (H3N2) vaccine (Charles River Laboratories) prior to injury and challenged with homologous (same strain, H3N2) or heterologous (different strain, H1N1) viruses to define whether injury alters virus-specific memory cells. Briefly, 8- to 10-week old mice were immunized by intramuscular injection into the tibialis inferior muscle of each limb with a total dose of 10 μg of vaccine, given in two injections (each 5 μg in 50 μl of PBS). A booster dose was administered into the same muscle 2 weeks later. Serum was collected 2 weeks after the second dose and assayed for virus-specific IgG by ELISA (as described above) prior to SCI. Seven weeks post-injury vaccinated injured and uninjured mice were infected intranasally with 1x10^4^ PFU of the same strain of live virus as the vaccine (H3N2) and monitored daily for survival. Since these studies were longer in duration compared to the primary infection model, age-matched non-vaccinated injured and uninjured mice were also infected as secondary controls. Virus-specific antibodies were monitored 10 and 30 days post-infection by IgG ELISA and neutralization assay. Surviving mice in the vaccinated groups were further challenged intranasally a month later with high dose (1x10^4^ PFU) of heterologous virus (H1N1, PR8) to test whether these mice were able to recover from infection with a new strain of virus.

### Statistical analyses

ANOVA, with Bonferroni post-test, was used to compare differences between groups. A Student’s *t* test was used to compare each time point within each group. Comparison of survival curves following infection was performed using the log-rank (Mantel-Cox) test. GraphPad Prism (GraphPad Software, La Jolla Ca, USA) was used for statistical analysis. Statistical significance was inferred when *p* ≤ 0.05.

## Results

### Impaired viral clearance increases mortality in chronically injured mice

**Fig. 1:**
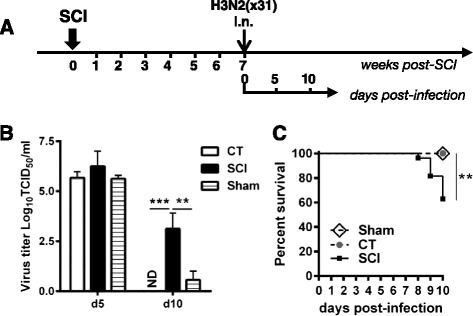
Impaired viral clearance in spinal cord injured mice. **a** Schematic of the experiment. Mice received a spinal cord injury (SCI) or laminectomy (Sham) at thoracic level T9 and 7 weeks later were infected intranasally with 1 × 10^4^ H3N2 (x31) influenza particles. An age-matched uninjured control group (CT) and a group receiving only a T9 laminectomy without injury were also infected and served as controls. Lungs were harvested 5- or 10-day post-infection for virus titer. **b** Viral titers were determined from lung homogenates isolated on day 5 and 10 post-infection. While all control uninjured mice cleared the virus, chronic SCI mice still had active virus in their lungs (mean ± SEM, 3 mice/group d5 and 10 mice/group d10, two-way ANOVA, **p < 0.01). Most of sham-operated mice had cleared the virus by day 10 (*n* = 8). *ND* not detectable. **c** Cumulative survival curves showed a significant increase in mortality among mice receiving a spinal cord injury compared to CT or Sham. Twenty-four to twenty-seven mice per group for CT and SCI, 8 mice/group for Sham, log-rank test, **p < 0.01

Infection with 1x10^4^ PFU dose of H3N2 in immunocompetent C57BL/6J mice establishes a robust influenza-specific immunity that clears virus by 10-day post-infection (dpi) [28]. In Fig. 1, we report the effect of virus infection with respect to viral titers and survival of SCI mice in comparison to sham-operated and uninjured control (CT) mice. The data presented in Fig. 1b demonstrates that viral infection was established uniformly 5 dpi in the lungs of all three groups. No statistical differences in viral titer were observed between groups (CT 5.67 ± 0.3, SCI 6.25 ± 0.7, Sham 5.62 ± 0.17, Log_10_TCID_50_/ml). However, on 10 dpi, while all CT mice cleared the virus, seven out of ten mice in the SCI injured group did not clear the virus and continued to have high titers of infectious virus in their lungs (3.13 ± 0.8 Log_10_TCID_50_/ml). Most of the sham-operated controls (6/8) cleared the virus, and both animals had significantly lower virus titer than the SCI group (Sham 0.5625 ± 1.24, SCI 3.13 ± 0.8 Log_10_TCID_50_/ml) indicating that the defect in viral clearance was due to spinal cord injury. Survival of mice (*n* = 27 SCI, *n* = 24 CT, *n* = 8 Sham) after virus infection was monitored in a second experiment for 10 days. As shown in Fig. 1c, 38.2 % of SCI mice succumbed to infection (*p* < 0.001, log-rank Mantel-Cox test).

### Maintenance of innate response in chronic SCI mice

**Fig. 2:**
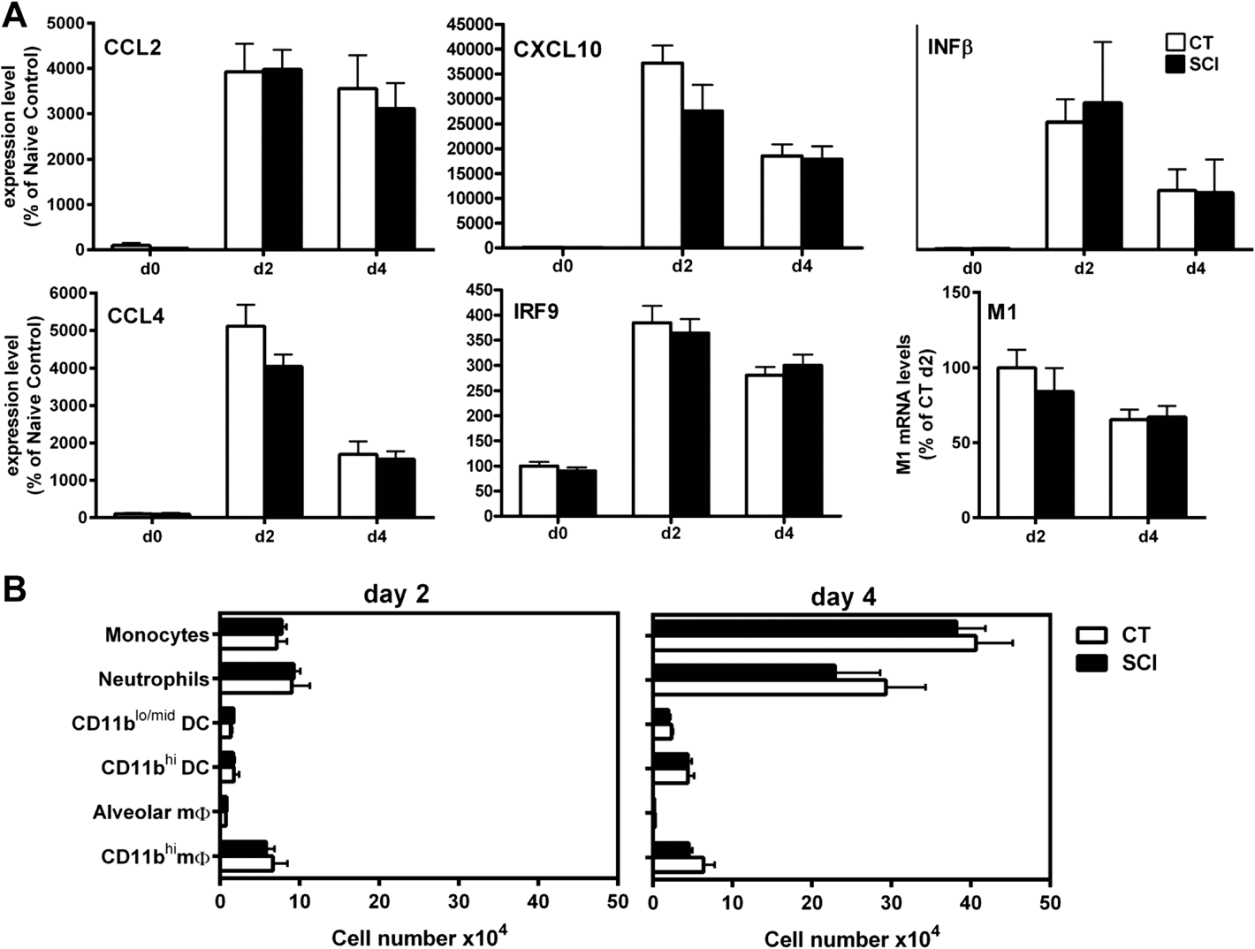
Early response to influenza in the lungs of spinal cord injured mice is not altered. **a** Expression of proinflammatory cytokines (CCL2, CXCL10, CCL4) and antiviral genes (IFNβ and IRF9) was examined in the lungs of chronic SCI and uninjured control mice prior to infection (d0) and 2- and 4-day post-infection using quantitative real-time PCR. Data are expressed as percent of uninjured control at d0 (mean ± SEM, 4 mice/group). In addition, we assessed the levels of virus using qPCR for the matrix 1 (M1) influenza gene at d2 and d4 post-infection. Data represents mean ± SEM (4 mice/group). **b** Innate immune response in the lungs of both CT and chronic SCI H3N2-infected mice was assessed by flow cytometry using the differential expression of markers F4/80, CD11b, CD11c, and Gr1 according to Hall et al. [22]. *Bars* represent the mean ± SEM (4 mice/group)

The innate immune system is considered as the first line of defense against viral infections. At the initial stage of infection, innate immune mechanisms in the lungs initiate antiviral responses including expression and secretion of cytokines and chemokines subsequent to attachment of viral particles to specific pattern recognition receptors [29]. The release of proinflammatory mediators recruits a rapid influx of neutrophils, monocytes, and natural killer (NK) cells to the site of infection and is pivotal for clearance of virus-infected cells [30]. To begin investigating the innate immune responses after influenza infection, we used qPCR to examine mRNA expression of antiviral genes, cytokines, and chemokines from the lungs, of infected chronic SCI mice and aged-matched controls at 0, 2, and 4 dpi. We chose the following genes based on their known activity in the antiviral response: interferon beta (IFNβ), interferon regulatory factor 9 (IRF9), monocyte chemotactic protein 1 (CCL2/MCP1), interferon gamma-induced protein 10 (CXCL10/IP-10), and macrophage inflammatory protein-1β (CCL4/MIP-1β) [31]. There was no statistical difference in the levels of gene expression of any of the cytokine/chemokine between chronic SCI and control groups (Fig. 2a). We also show that the virus-specific M1 gene (matrix) is expressed in the lungs of CT and chronic SCI mice confirming that there was no impairment of establishment of virus infection (Fig. 2a).

The composition of innate immune cells was assessed on 2 and 4 dpi from lung lavage of individual mice. In response to H3N2 influenza infection, both uninjured and chronic SCI mice had an increase in the appearance of innate immune effector cells including neutrophils, alveolar macrophages, and myeloid-derived dendritic cells (DC) into the airway as reflected by the numbers of cells present over time (Fig. 2b). By far, the largest number of cells were neutrophils and monocytes (CD11b^+^CD11c^-^) followed by the conventional DC (CD11^+^CD11c^+^), macrophages (F4/80^+^ and CD11b^high^), and alveolar macrophages (CD11c^high^/CD11b^low^). The ability of all of these cell types to control viral replication and the development of adaptive immunity is well established [32]. Thus, virus-specific innate immunity within the lungs of chronically injured mice is not compromised.

### Reduction in virus-specific antibodies in chronic SCI mice

**Fig. 3:**
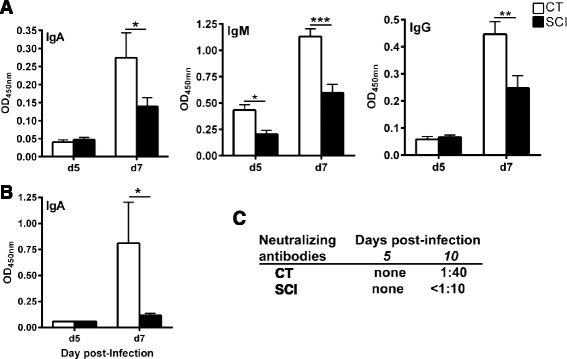
Impaired antibody response to influenza in chronic SCI mice. **a** Influenza virus-specific antibody response was assessed by ELISA using H3N2 virus-coated plates and diluted sera from chronic SCI and CT mice 5 or 7 days post-infection. *Bar graphs* represent the mean ± SEM, 5 mice/group. Data was normalized after subtracting the values from uninfected mice from both the uninjured and injured groups at day 0 prior to infection. **b** Virus-specific IgA response was measured in the BAL of infected mice on day 5 and day 7. Mean ± SEM, 5 mice/group. **c** The ability of the antibodies to neutralize the virus was tested using serial dilutions of the serum from uninjured CT and SCI mice

Virus-specific antibodies and cytotoxic CD8^+^T cells are key components of the adaptive immune that are necessary to clear virus infection [33]. These antibodies are rapidly induced early during primary infection [34]. Mucosal IgA generated in the respiratory tract prevents virus attachment, whereas systemic IgG is required to prevent infection either by neutralization of virus infectivity or antibody-dependent cell-mediated cytotoxicity (ADCC) or complement-mediated lysis [35]. Various isotypes of virus-specific antibodies were analyzed in CT and chronic SCI mice using standardized ELISA. Chronic SCI mice demonstrated a significant reduction in virus-specific IgM on 5 dpi (*p* < 0.05) and 7 dpi (*p* < 0.001) post-infection in the serum compared to uninjured CT (Fig. 3a). In addition, both IgG and IgA were also diminished in chronic SCI mice (Fig. 3a) on 7 dpi. Similarly, mucosal IgA responses in SCI mice were reduced significantly (Fig. 3b) in comparison to CT. Individual sera were further tested using a virus neutralization assay to evaluate whether virus-specific antibodies were able to block virus infection. Negligible neutralizing activity was seen early in infection on day 5 in both groups. However, on 10 dpi, all of the mice from the uninjured CT group had a virus neutralization titer of 1:40 whereas none of the sera isolated from chronic SCI mice had neutralizing activity (Fig. 3c). In the chronic SCI group, neutralization was not detected at a serum dilution of 1:10, which is the limit of detection for this assay.

### Splenic B cells from chronic SCI mice are functional

**Fig. 4:**
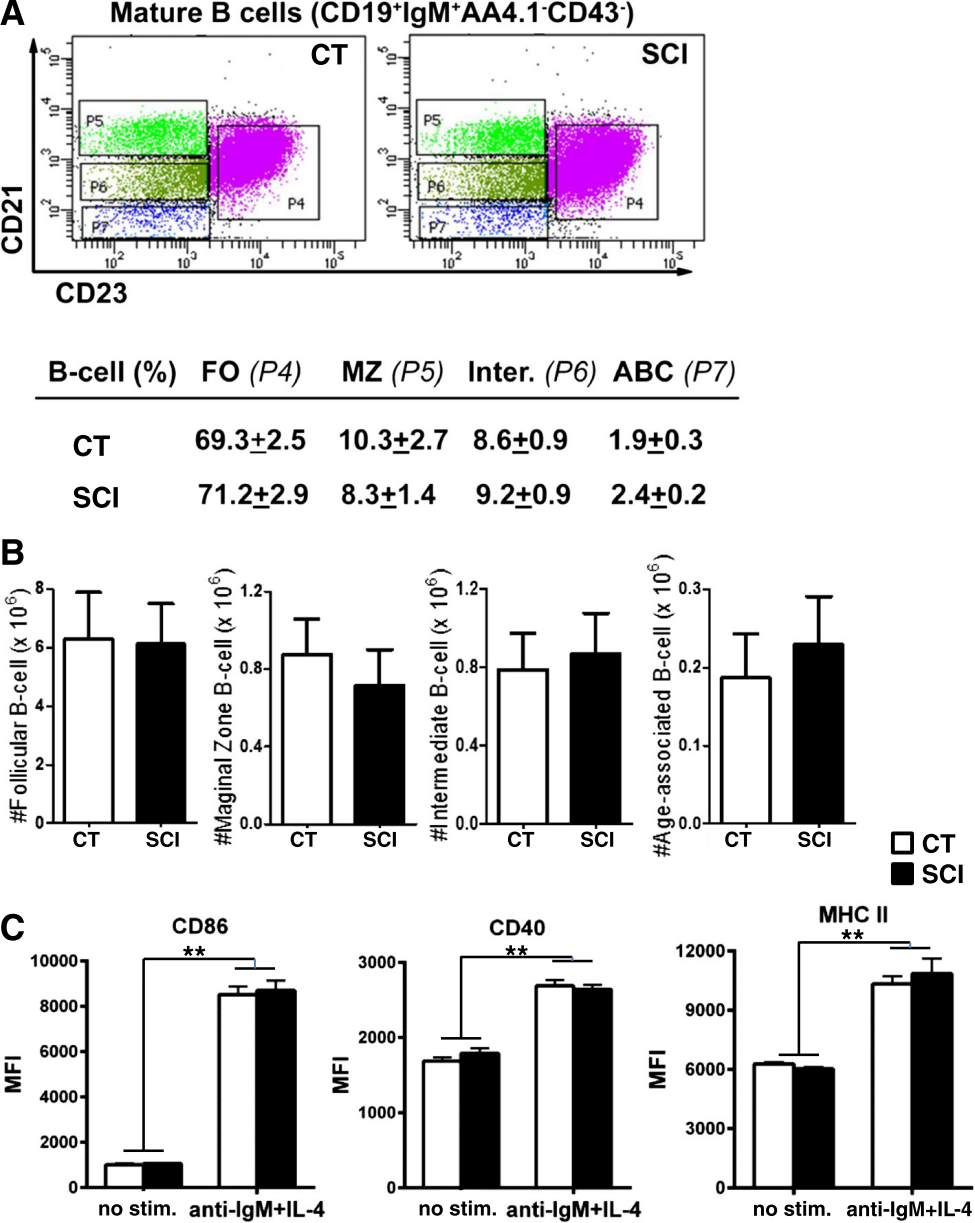
Intrinsic function of B cells is preserved in chronically injured mice. **a**, **b** The frequency and number of splenic mature B cell subtypes in chronic SCI mice (SCI) are not statistically different from uninjured mice (CT). **a** Splenocytes from chronic SCI mice were isolated 7-week post-injury along with age-matched uninjured CT. Mature B cells were gated as CD19^+^IgM^+^AA4.1^−^CD43^−^ B cells and then further subdivided into follicular (FO, CD23^+^CD21^+^), marginal zone (MZ, CD23^−^CD21^hi^), intermediate (Inter., CD23^−^CD21^lo^), and age-associated B cells (ABC, CD23^−^CD21^−^). Data in the table represent the % of B cells in the different subsets (mean ± SEM, *n* = 4/group). **b** *Bar graphs* represent the total cell number for each subset of B cells (mean ± SEM, *n* = 4/group), calculated from the percentages obtained in A. **c** Functional in vitro assay on isolated splenic B cells from chronic SCI and uninjured CT mice. Cells were stimulated with anti-IgM and IL4 or left unstimulated. B cell activation was analyzed by the surface expression of CD86, CD40, and MHCII markers by flow cytometry, and the data is represented as mean fluorescence intensity (MFI)

Since we showed a deficit in a humoral immune response with reduced virus-specific antibody production and neutralization activity, we tested the possibility that B cells from chronic SCI mice are functionally impaired 7-week post-injury before infection. First, we examined the distribution of B cells into different subsets, (follicular (FO), marginal zone (MZ), intermediate (Inter.), and age-associated B (ABC) cells) by flow cytometry (Fig. 4). We did not find any significant differences in either the frequency or the number of these B cells (Fig. 4a, b). We next assessed their function in vitro by stimulating them with both anti-IgM and IL4, which mimics BCR-dependent activation (Fig. 4c). As expected following stimulation, expression of CD86, CD40, and MHCII was significantly increased compared to unstimulated controls. However, activation levels between CT and chronic SCI B cells were not statistically significant suggesting that B cells from injured mice are not intrinsically impaired. This further suggests that the impairment observed in vivo may be due to deficits in antigen presentation and/or CD4^+^T helper cells [36].

### Decrease in virus-specific CD8^+^T cells in injured mice

**Fig. 5:**
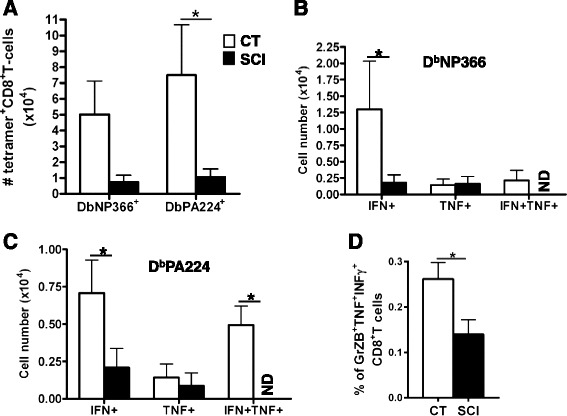
Spinal cord-injured mice produce significantly less virus-specific CD8^+^T cells than CT mice. **a** Splenocytes from naïve and SCI mice isolated 7-day post-infection were analyzed by flow cytometry using tetrameric complexes of either the nucleoprotein peptide (NP_366–374_) or the polymerase 2 protein peptide (PA_224–233_) presented by class I H-2D^b^ (mean ± SEM, 4–5 mice/group, *p < 0.05). **b**, **c** In vitro intracellular cytokine assay revealed a significant decrease in the number of CD8^+^T cells producing IFNγ following stimulation with the peptide D^b^NP366 (**b**) and a significant decrease in the number of CD8^+^T cells producing IFNγ as well as both IFNγ and TNF following stimulation with the peptide D^b^PA224 (**c**). **d** Percentage of CD8^+^T cells expressing granzyme B, TNFα, and IFNγ following stimulation with peptide D^b^PA224. *Bar graphs* represent the mean ± SEM, *n* = 4–5 mice/group, *p < 0.05

CD8^+^T cells (cytotoxic T lymphocytes, CTL) play an important role in influenza-infected lungs by destroying viral-infected cells via perforin/granzyme-dependent granule exocytosis as well as by FasL/Fas-mediated apoptosis following T cell receptor (TCR) engagement [37]. Secretion of proinflammatory cytokines (e.g., IFNγ and TNFα) is hallmarks of these CTLs which contributes to the recruitment and activation of innate inflammatory cells, in particular, inflammatory CD11c^hi^ DC and pDC [38]. The infectious virus recovered from infected injured mice on day 10 (Fig. 1b) indicates that CTL response to influenza antigens may also be compromised in chronic SCI. We therefore quantified the number of virus-specific CD8^+^T cells and their ability to produce intracellular cytokine IFNγ and/or TNFα day 7 after infection. Figure 5a demonstrates that chronic SCI mice had a significant (*p* < 0.05) reduction of virus-specific CD8^+^T cells of 6.5-fold in PA-specific cells in the spleen compared to infected uninjured controls. Importantly, the total numbers of CD8^+^T cells recovered from both groups of mice were not significantly different (CT 3.9 ± SD1.6; SCI 3.0 ± SD2.0; x10^6^) and therefore recruitment of these cells in response to infection was not defective. Next, the ability of these CD8^+^T cells to secrete antiviral cytokines was analyzed as a qualitative measure of virus-specific CD8^+^T cell function by intracellular cytokine assay after in vitro stimulation in culture for 6 h with virus-specific MHC class I restricted peptides NP_366–374_ and PA_224–236_. The numbers of CD8^+^T cells producing IFNγ or both IFNγ and TNFα were significantly reduced or were not detectable in chronic SCI mice compared to CT (Fig. 5b, c). Furthermore, the percentage of PA_224–236_ virus-specific CTL producing granzyme B, an effector molecule to initiate cell death in virus-infected cells was also significantly reduced in chronic SCI compared to uninjured controls (Fig. 5d).

### Vaccination prior SCI generates protective immunity

**Fig. 6:**
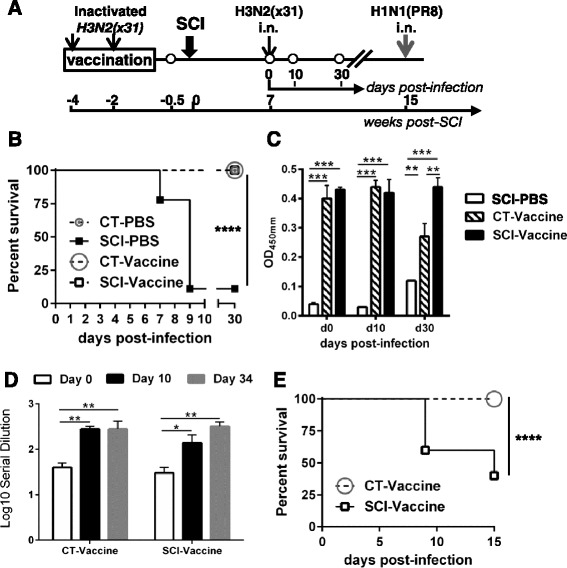
Vaccinated chronic SCI mice are protected from a strain-matched influenza virus but not from a second challenge with a different strain. **a** Schematic of the experiment. Mice were divided into two groups (*n* = 15/group): one group received two doses of inactivated H3N2 vaccine and the other PBS 1 month prior to SCI. The two groups were further divided into two sub-groups, one receiving a spinal cord injury at thoracic level T9 (SCI, *n* = 10) and the other was not injured (CT, *n* = 5). Seven weeks post-injury, all mice were infected with H3N2 (HKx31) by intranasal route. Blood samples (*open circles*) were collected 4 days before SCI to confirm that all vaccinated mice had developed influenza-specific antibody titer and at day 0, 10, and 30 post-infection. The two groups of vaccinated mice (CT-vaccine, *n* = 5 and SCI-vaccine, *n* = 10) were challenged a second time with a different influenza strain, H1N1 (PR8). **b** Survival curve for the four groups of mice (*n* = 5/group for CT and 10/group for SCI) infected with H3N2 (HKx31). Only the chronic SCI mice that were not vaccinated (SCI-PBS) showed a dramatic decrease in survival. ****p < 0.0001, log-rank (Mantel-Cox) test. **c** The virus-specific antibody response in vaccinated SCI mice was similar to that of the vaccinated CT group (*n* = 5). **p < 0.01, ***p < 0.001. **d** Chronic SCI mice previously vaccinated produced neutralizing antibodies to the virus to the same extent as uninjured CT mice. *p < 0.05, **p < 0.01. **e** Vaccination of chronic SCI mice does not protect against a second challenge with a different influenza strain. Survival curves of H3N2-vaccinated uninjured CT (*n* = 5) and chronic SCI mice (*n* = 10) challenged with a dose of H1N1 virus. ****p < 0.0001, log-rank (Mantel-Cox)

Our data show that adaptive immunity against influenza virus is severely attenuated in chronic T9 SCI mice during primary infection. Therefore, we asked the question whether antigen-specific memory obtained through vaccination prior to spinal cord injury would protect mice against a homologous viral infection. This has clinical implications since SCI patients are either vaccinated prior to injury or will receive yearly flu vaccine as a standard of care to prevent flu-associated morbidity and hospitalization [39]. As shown in Fig. 6a, mice were injected intramuscularly with two doses of inactivated H3N2 (HKx31) virus before sustaining a spinal cord injury at thoracic level T9. Seven weeks later, mice were challenged with the same virus and monitored daily for survival until day 30 post-infection. All vaccinated chronic SCI mice (SCI-Vaccine) survived influenza infection to the same extent as vaccinated uninjured CT mice (CT-vaccine) and non-vaccinated uninjured CT mice (CT-PBS) (100 % survival, Fig. 6b). This was in sharp contrast to non-vaccinated chronic SCI mice (SCI-PBS) where survival rate dropped to 77 % (day 7) and 11 % (day 10).

We next analyzed sera for virus-specific IgG production and neutralizing activity from all groups to confirm vaccine responses. Sera from non-vaccinated chronic SCI mice (SCI-PBS) had low levels of virus-specific IgG and did not neutralize virus infectivity compared to uninjured controls (data not shown). In contrast, vaccinated SCI group (SCI-Vaccine) produced a similar amount of virus-specific IgG as the vaccinated CT mice (Fig. 6c). Importantly, chronic SCI-vaccine mice had high levels of virus neutralizing activity (Fig. 6d) similar to the CT-vaccine group, which correlated with their 100 % survival.

### Spinal cord injury impairs the virus-specific memory T cell response

Vaccination with an inactivated virus is designed to generate protective antibodies primarily against the hemagglutinin (HA) surface protein of the viral particle [40]. However, influenza viruses undergo genetic mutations such as drift or shift that contribute to the generation of viruses that can escape neutralizing antibodies, or give rise to new viruses to which, there is no pre-existing antibodies (leading to a potential pandemic) [41]. In these instances, cross-reactive memory T cells targeted to conserved internal proteins can provide immune protection between two different virus strains [42]. In order to investigate whether the virus-specific compartment-cell memory was functional after chronic SCI, we re-challenged both CT and SCI vaccinated groups 2 months after the H3N2 (HKx31) infection with a high dose (1x10^4^ PFU) of H1N1 (PR8) virus by intranasal infection. H3N2 and H1N1 viruses share the same internal proteins and generate robust CD8^+^T cell immunity against a number of viral peptides. In immunocompetent mice, these memory T cells mediate cross-reactive memory T cell responses that clear the virus earlier [43]. As shown in Fig. 6e, while CT mice were protected against a second challenge, only 60 % of chronic SCI mice survived at day 9 post-infection and 40 % at day 15. High virus titers were recovered from the lungs of mice succumbing to infection (data not shown). Thus, cross-reactive T cell memory responses to a heterologous challenge was impaired after chronic SCI indicating that injury can also permanently damage memory-specific T cell responses to new influenza virus infections.

## Discussion

Patients with SCI live for years and suffer from significant morbidity due to recurrent respiratory infections. We therefore hypothesized that chronic immune paralysis after SCI would impair virus-specific host immunity. Using both primary and secondary infection models of influenza infection, we report this study is the first to demonstrate that thoracic T9 spinal cord injury alters the adaptive immune response directed against a clinically important influenza A respiratory virus.

Crosstalk between the nervous system and the immune system plays a pivotal role in maintaining homeostasis of the host. This coordination is achieved via the sympathetic nervous system (SNS) and the hypothalamic-pituitary-axis (HPA), which regulates a variety of cytokines, hormones, and neurotransmitters [44, 45]. Thus, direct injury to the neural pathways innervating the spinal cord contributes to functional deficit in the peripheral immune response and renders patients with SCI more susceptible to viral and bacterial infections [46]. Indeed, patients with longer duration of SCI have higher frequency of respiratory infections, which increases with aging [14]. We report here for the first time that chronic SCI alters the adaptive immunity to influenza, an important human respiratory virus, in a thoracic level T9 spinal cord contusion model.

### Level of injury

We used a thoracic T9 level of injury for the following reasons. First, several studies looking at the effects of SCI on the immune system have compared high thoracic (T3) versus low thoracic (T9) level of injuries and found that T3 injury has a greater disruptive effect on the HPA and SNS axis than a lower thoracic level T9 [6] with T3 injury being more detrimental to B cell function compared to T9 [4, 9, 10]. Despite those differences, mice sustaining T9 SCI were as sensitive to mouse hepatitis virus (MHV) infection and mortality as T3 spinal cord injured mice [4]. Second, we showed in our previous study that mice sustaining a lower thoracic T9 SCI display deficits in T cell function 7-week post-injury accompanied by increased levels of splenic norepinephrine that contributed to the exhausted phenotype and dysfunction of those T cells [11]. Third, T3 injury will affect respiratory capacity to a greater extent than T9 injury [47] and would add another layer of complexity in the influenza infection model. Lastly, patients with lower thoracic injury are more active and may be more exposed to pathogens, which would increase their susceptibility to respiratory viruses such as influenza transmitted by aerosolized contact.

### Influenza infection

The immune response to influenza infection provides a model to systematically study the contributions of host immunity by addressing both innate and adaptive compartments. We demonstrate that primary infection with influenza A virus of chronically injured mice (7-week post-SCI) at thoracic level T9 causes severe morbidity and mortality. Approximately 40 % of injured mice succumbed to infection (Fig. 1c) and 60 % of the injured mice had high viral titers in their lungs on 10 dpi when compared to uninjured control or sham-operated animals (Fig. 1b). Our data are in agreement with the study done by Held and colleagues [4] where increased mortality was observed in T9-injured mice infected with a mouse hepatitis virus (MHV), 4-week post-injury compared to uninjured control mice. However, in contrast to our study, an increase in MHV titer in the liver of SCI group was observed early in infection in comparison to uninjured mice. We did not find any significant difference in viral load on day 5 during the establishment of virus replication (Fig. 1b) in injured, uninjured, and sham mice, which is indicative of SCI injury not exacerbating the viral infection per se. This was also confirmed by the ability of chronic SCI mice to mount an innate immune response as demonstrated by the significant increase in the expression of antiviral genes and the presence of innate immune effector cells in the airways of both influenza virus-infected CT and chronic SCI mice (Fig. 2a, b).

The adaptive immune system comprising of humoral and T cell-mediated immunity plays a crucial role in controlling respiratory virus infections such as influenza [17]. The virus-specific antibodies are targeted to two outer surface glycoproteins namely hemagglutinin (HA) and neuraminidase (NA) both of which are predicted to correlate with protection [48] and recovery from lethal infections [49]. The main antibody isotypes produced during influenza infection are IgA, IgM, and IgG in serum with secretary IgA being more prominent at the local site of replication [17]. Influenza-specific IgM initiates neutralization of the virus by complement-mediated system, and IgG is required for generation of long-lived protective antibodies. Mucosal IgA is required for neutralizing the virus at the site of replication and mediating cross-protection against drift viruses with the same subtype [50]. Thus, cumulative deficits in all virus-specific isotypes seen as early as 5 dpi in chronic SCI mice may contribute to the impaired viral clearance on 10 dpi. Surprisingly, there was no difference between chronic SCI and uninjured control mice in the percentages of various subsets of B cells or their activation following in vitro stimulation prior to infection indicating no intrinsic impairment in B cell function and pointing to a possible deficit in CD4^+^T helper function to thymus-dependent responses and germinal center reaction after infection. The requirement of CD4^+^T cell helps for thymus-dependent responses, and germinal center reaction during primary infection [51, 52] is well known and merits further investigation in this chronic SCI model.

The importance of virus-specific CD8^+^T (CTLs) cells during influenza infection is also well documented [37, 53, 54]. During primary infection, CTLs traverse to the site of infection and eliminate virus-infected targets and upon re-infection with different subtypes of influenza viruses; these CTLs can provide cross-protection as their specificity is generally derived from conserved internal viral proteins [55]. In chronic SCI mice, CD8^+^T cells restricted to conserved proteins of the virus were reduced both quantitatively and qualitatively in spleens during primary infection in comparison to CT animals. The repertoire of influenza CTLs in response to human infection is also targeted to these internal proteins [56], and therefore understanding these mechanisms in mouse infection models of SCI will allow us to design targeted therapy. Mechanistically, our data imply that injury may alter the presentation of viral antigens by splenic antigen presenting cells to CD8^+^T cells located at the T cell zones. One can also infer from our published work that accumulation of norepinephrine within the spleen at the chronic phase promotes immune suppression as both T and B cells express neuroendocrine receptors [11]. Future studies are in progress to test these hypotheses.

### Vaccination and challenge with homologous and heterologous influenza virus strains

We next asked the question whether intramuscular vaccination with whole inactivated H3N2 virus administered before injury would protect chronic SCI mice from a subsequent homologous infection. Our results show that previously vaccinated chronic SCI mice are protected from a secondary challenge with the same influenza strain, displaying high levels of virus-specific antibodies that neutralize virus infectivity and control mortality. Likewise, in a mouse model of chronic SCI, Oropallo and colleagues demonstrated that secondary humoral immunity remains functional and can be boosted after injury [10]. Collectively, our data confirms that pre-existing vaccine-specific B cell memory does not require neuronal regulation, is refractory to CNS-mediated changes of HPA axis, and thus has implication for influenza vaccination in SCI patients. In contrast, memory-specific CD8^+^T cells generated after injury were compromised in chronic SCI mice heterologous virus challenge model affirming that injury causes permanent damage to the peripheral immune response to pathogen-specific immunity even at T9 level. This observation also provides us with further opportunity to assess the role of CD4^+^T cells in this model in the future, as it is known that in comparison to primary infection, CD4^+^T cells play a pivotal role on the size and magnitude of CD8^+^T cells during a recall response [52, 57].

## Conclusions

We conclude that virus-specific adaptive immune response is severely hampered in chronic SCI mice using a mouse model of influenza infection. Furthermore, we provide evidence that chronic SCI also affects the formation of immunological memory in a secondary virus infection model. To our knowledge, this is the first comprehensive analysis of innate and adaptive immune response against a clinically relevant respiratory pathogen in a chronic SCI preclinical model. A concerted effort is underway to analyze susceptibility to infections in patients using the National Spinal Cord Injury Database [58]. Thus, future research using virus infection models will allow us to dissect mechanisms of how injury to the CNS weakens peripheral immunity and whether specific treatments can be designed to improve outcome of SCI patients following infection.

## Additional file

Additional file 1: Table S1. Primer sequences for qRT PCR. (PDF 146 kb)

## Abbreviations

BAL: bronchoalveolar lavage
CIDS: CNS injury-induced immunodepression
ELISA: enzyme-linked immunosorbent assay
FBS: fetal bovine serum
HRP: horseradish peroxidase
i.m.: intramuscularly
i.n.: intranasally
i.p.: intraperitoneally
MDCK: Madin-Darby canine kidney
PBS: phosphate-buffered saline
PCR: polymerase chain reaction
SCI: spinal cord injury
