# Supplementary material for: Chronic spinal cord injury attenuates influenza virus-specific antiviral immunity
Source: J Neuroinflammation. 2016 May 31;13:125. doi: 10.1186/s12974-016-0574-y (PMC4886448; doi:10.1186/s12974-016-0574-y)
Supplement: Additional file 1: Table S1. — Primer sequences for qRT PCR. (PDF 146 kb) [file 12974_2016_574_MOESM1_ESM.pdf]

Table 1. Primer sequences for qRT PCR

Gene	Forward Primer	Reverse Primer	Tm (°C)	Product (bp)
**IFNβ**	5'-ATGACGGAGAAGATGCAGAAGAGT	5'-TCATCCAGGAGACGTACAACAATA	52.3	134
**IRF9**	5'-AGAGCAGCATGGAGCAGGTGGAGT	5'-ATGGGGCAAAGGCGCTGAACAAAG	59.1	136
**CCL2**	5'-CCCCACTCACCTGCTGCTAC	5'-CCTGCTGCTGGTGATCCTCTT	52.9	86
**CCL4**	5'-GTGGCTGCCTTCTGTGCTC	5'-GATCTGTCTGCCTCTTTTGGTCA	55.6	180
**CXCL10**	5'-GCCGTCATTTTCTGCCTCATCCT	5'-CTCATTCTCACTGGCCCGTCATC	57.2	113
**M1**	5'-AGCCGAGATCGCGCAGAGACT	5'-TGAGCGTGAACACAAATCCTAAAA	53.4	137
**β-actin**	5'-ATGGTGGGAATGGGTCAGA	5'-CACGCAGCTCATTGTAGAAGG	54.8	157
